# A COVID-19 Hotspot Area: Activities and Epidemiological Findings

**DOI:** 10.3390/microorganisms8111711

**Published:** 2020-10-31

**Authors:** Francesca Cito, Laura Amato, Alessandra Di Giuseppe, Maria Luisa Danzetta, Simona Iannetti, Antonio Petrini, Alessio Lorusso, Barbara Bonfini, Alessandra Leone, Romolo Salini, Adamo Mancinelli, Giuseppe Torzi, Giovanni Savini, Giacomo Migliorati, Thomas Schael, Nicola D’Alterio, Paolo Calistri

**Affiliations:** 1 Istituto Zooprofilattico Sperimentale dell’Abruzzo e del Molise G. Caporale, 64100 Teramo, Italy; l.amato@izs.it (L.A.); a.digiuseppe@izs.it (A.D.G.); m.danzetta@izs.it (M.L.D.); s.iannetti@izs.it (S.I.); a.petrini@izs.it (A.P.); a.lorusso@izs.it (A.L.); b.bonfini@izs.it (B.B.); a.leone@izs.it (A.L.); r.salini@izs.it (R.S.); g.savini@izs.it (G.S.); g.migliorati@izs.it (G.M.); n.dalterio@izs.it (N.D.); p.calistri@izs.it (P.C.); 2 Lanciano-Vasto-Chieti Local Health Unit, 66100 Chieti, Italy; adamo.mancinelli@asl2abruzzo.it (A.M.); giuseppe.torzi@asl2abruzzo.it (G.T.); direzione.generale@asl2abruzzo.it (T.S.)

**Keywords:** Abruzzo, coronavirus, COVID-19, hotspot, Italy, SARS-CoV-2, seroprevalence

## Abstract

By late March 2020, Villa Caldari, a small village of the municipality of Ortona (Abruzzo region), was registering an incidence rate of COVID-19 cases ten times greater than the overall municipality and was declared a hotspot area. Twenty-two days later, epidemiological investigation and sampling were performed, to evaluate SARS-CoV-2 circulation and the presence of SARS-CoV-2 antibodies. Overall, 681 nasopharyngeal swabs and 667 blood samples were collected. Only one resident of the village resulted in being positive for RNA viral shedding, while 73 were positive for SARS-CoV-2 antibodies. The overall seroprevalence was 10.9%. The difference between the seroprevalence of infection in asymptomatic and symptomatic individuals was significant (χ^2^ = 14.50 *p*-value = 0.0001). Amongst the residents positive for antibodies, fatigue and/or muscle pain, fever and anosmia were the most experienced symptoms, whose most frequent onset was observed during the first two weeks of March. Familial and habit-related clusters were highlighted. Nevertheless, the investigations showed a low SARS-CoV-2 circulation in the village at the time of the sampling, demonstrating virus transmission could be limited when strict emergency measures are followed. Given the favorable results, the emergency measures were then lifted.

## 1. Introduction

The novel human coronavirus, named severe acute respiratory syndrome coronavirus 2 (SARS-CoV-2) [1,2], was officially identified for the first time in Wuhan, China, in late December 2019 [3]. SARS-CoV-2 is the causative agent of the coronavirus disease 2019 (COVID-19) [4] that was declared a pandemic by the WHO on 11 March 2020 [5,6]. As a coronavirus, the 5′-most two thirds of the genome comprises the ORF1ab gene, which consists of two overlapping open reading frames, ORF 1a and 1b. Located downstream of ORF1b are 4 ORFs that code for a common set, to all CoVs, of structural proteins (spike (S), envelope (E), membrane (M) and nucleocapsid (N) proteins). Among them, S protein plays the most important roles in viral attachment, fusion and entry, and it serves as a target for development of antibodies, entry inhibitors and vaccines. SARS-CoV-2, like the earlier SARS-CoV that emerged in 2002/2003, uses the receptor binding domain (RBD) of the viral S1 portion of the spike (S) protein to bind to the angiotensin-converting enzyme 2 receptor that is widespread in epithelial and endothelial cells of the respiratory and gastrointestinal tracts of mammals [2].

On February 21, the first Italian autochthonous confirmed COVID-19 case was identified in Lombardy [7,8]. In late February 2020, Italy witnessed a rise in SARS-CoV-2 infections and became one of the first pandemic hotspots amongst Western countries [9]. Indeed, in a few days, an increasing number of cases was identified, some of which not having any contact with the first patient or with any known confirmed COVID-19 case. As a consequence, a lockdown of ten hotspot municipalities in the northern regions of Lombardy and Veneto was enforced by the Italian Government [8,10]. As a growing number of COVID-19 cases started to be also identified in the other regions, from 10 March the lockdown was extended to the whole country [10]. Emergency measures such as restriction on mobility, social distancing and the closure of all nonessential services, were applied. COVID-19 cases were mainly detected in Northern Italy, where less than three weeks after the notification of the first outbreak, the cases were overloading the local hospitals [7]. Given the impact of the pandemic, the Italian Ministry of Health appointed the Istituti Zooprofilattici Sperimentali (IIZZSS) to support the National Health Care system in testing the nasopharyngeal swabs for SARS-CoV-2 RNA [7].

Overall, fewer cases were notified in Regions of Central and Southern Italy [11]. In Abruzzo region, Central Italy, the first COVID-19 case was confirmed on February 27 in a male patient originating from Lombardy [7].

The first COVID-19 fatality occurred on the 5 March, involving a male patient resident in “Villa Caldari”, a small village of about 900 inhabitants located in the municipality of Ortona, Chieti province, Abruzzo region (42°17′43.9″N, 14°21′37.6″E). The case was also the first to be detected in Villa Caldari, and twenty more cases, with eight fatalities, were confirmed in the following weeks, representing 53.5% of all cases reported in the whole municipality of Ortona (around 23,000 inhabitants). Considering the small population of the village, its incidence rate (×100,000 inhabitants) was ten times greater than the overall Ortona municipality [12]. In light of the alarming epidemiological situation, the President of Abruzzo Region declared Villa Caldari a hotspot area on 27 March [12]. Consequently, additional emergency measures were implemented in the village to limit the spread of SARS-CoV-2 [13]. Contingency measures included the ban on movements of people (within, from and to the village), and the stop of all non-essential activities. No one was allowed to leave the village: People could order doorstep delivery of groceries and essential items or collect them at delivery points.

Moreover, the Local Health Authority (LHA) of Chieti asked the Istituto Zooprofilattico Sperimentale dell’Abruzzo e del Molise “G. Caporale” (IZSAM) for a support in diagnostics and epidemiological investigations. IZSAM, in collaboration with the LHA of Chieti, conducted an epidemiological study on Villa Caldari’s population during the lockdown period. The goal was the evaluation of the viral circulation and the presence of antibodies against SARS-CoV-2 in the population. The study meant to support the Regional Authorities’ decisional process for the withdrawal of the emergency measures taken for the village. 

## 2. Materials and Methods 

### 2.1. Ethical Approval Statement

The testing of suspected COVID-19 cases, contacts and residents in Villa Caldari was conducted within the official surveillance programme established by the Italian Health Authorities and did not require ethical approval. Written informed consent was obtained from all participants.

### 2.2. Study Design 

On 18th and 19th of April 2020, one and a half months after the first COVID-19 case in Villa Caldari, and 22 days after the local lockdown came into force, a voluntary survey was carried out on the whole population of the village. The survey was conducted to investigate population exposure to SARS-CoV-2 before and during the lockdown and to evaluate the possible (partial or complete) withdrawal of the social restrictions. All residents were informed in advance by the municipality’s authorities about the date and place of sample collection. The survey was also advertised on local media. Two days of sampling were planned, and people were invited for testing according to family groups and home addresses. Healthcare personnel and law enforcement engaged in the maintenance of the emergency measures were involved in the survey. 

The area dedicated to sampling was organized with separate entrance and exit pathways, to maintain the biosecurity measures among individuals. At the arrival, people were registered upon the presentation of their national health ID card. During the registration, a brief interview was performed, and a form with personal data and epidemiological information was filled (see Supplementary materials). The collected information concerned occupation, household members and presence of relevant COVID-19 clinical signs. At the end of the registration process, two labels bearing a sequential number were generated, to univocally identify the nasopharyngeal swab and the blood sample collected for each person by the health personnel. The labeled samples were stored in insulated thermal bags. Additional door-to-door sampling sessions were organized for individuals unable to reach the sampling location, due to health reasons or because they were subjected to health surveillance and isolation for COVID-19. 

A second epidemiological enquiry was performed on 27 June, to collect additional information from people who tested positive at the first serological survey (see Supplementary Materials).

### 2.3. Laboratory Methods 

Molecular detection of SARS-CoV-2 RNA was conducted as previously described [14,15]. Briefly, two steps comprised the workflow for SARS-CoV-2 RNA detection. The first includes virus inactivation (PrimeStore^®^ MTM, Bethesda, MD, USA) in a BSL3 biocontainment laboratory, starting from a total volume of 200 μL of oropharyngeal (OF) swab transport medium and nucleic acid purification of MagMaxTM CORE (Thermo Fisher Scientific, Waltham, MA, USA), according to the manufacturer’s instructions. The second consists of RNA detection by the TaqManTM 2019-nCoV Assay Kit v2 (qPCR, Thermofisher, Waltham, MA, USA). This test targets three different portions of SARS-CoV-2 genome located in the replicase, S and N protein encoding genes, respectively. Results are interpreted according to manufacturer’s recommendation. Overall, samples showing C*_T_* > 37 are considered negative for SARS-CoV-2 RNA.

Serological analysis was performed starting from 10 µL of serum sample by means of the WANTAI SARS-CoV-2 Total Ab ELISA (Beijing Wantai Biological Pharmacy Enterprise Co., Beijing, China). This test, which is able to detect all Ab classes, is a two-step incubation antigen “sandwich” enzyme immunoassay kit, which uses polystyrene microwell strips pre-coated with a recombinant SARS-CoV-2 antigen. The antigen used in the assay is the RBD of SARS-CoV-2 S1 protein. The optical density is measured at 450 nm, and the results are calculated by relating each sample absorbance value (A) to the cutoff value (CO), obtained by multiplying the mean absorbance value for the negative controls by 2.1. The resulting A/CO values are <1.0 and ≥1.0, corresponding to negative and reactive results, respectively. The sensitivity of the Wantai Total Ab ELISA is equivalent to that of other CE-authorized ELISA tests at 93%. The specificity of the Wantai Total Ab ELISA is 100% compared to other commercially available ELISA tests [16].

### 2.4. Statistical Analyses 

The information gathered on the paper forms were copied into Microsoft Excel^®^ (Microsoft Corporation, 2013) spreadsheet. Data management was performed by using Microsoft Access^®^ (Microsoft Corporation, 2013) and statistical analysis by Statistical Software for Excel—XLSTAT (XLSTAT Version 2013.2.04 Copyright Addinsoft, 1995–2013). 

The possible existence of a relationship between the serological results and the age and the gender of tested persons was assessed by the calculation of a chi-square (χ^2^) test. A two-tailed Mann–Whitney test was applied to evaluate differences between the age of serologically negative and positive individuals. Family group members were divided according to their composition in three groups (single; 2–3; >3), and a chi-square (χ^2^) test was used to assess any difference between the serological positive response and the number of people belonging to each family group. 

The sampled population was also classified into two risk groups based on the employment declared. According to the classification (at risk or not at risk), a chi-square (χ^2^) test was undertaken to assess the possible association between the occupational categories and the presence of clinical symptoms; a chi-square (χ^2^) test was also used to evaluate the possible association between asymptomatic and symptomatic individuals and the presence of a positive antibody response.

## 3. Results

During the survey, out of 958 persons officially registered as residents in the village, 687 people were sampled (687/958, 71.7%): 643 on the sampling site and 44 through door-to-door activity. People sampled door-to-door answered to a shorter questionnaire; therefore, it was not possible to collect from these persons the whole set of epidemiological information. 

Both nasopharyngeal and blood samples were collected from 661 individuals. Twenty persons declined the blood sampling, and collection of nasopharyngeal swabs was not possible for six individuals. Therefore, an overall number of 681 nasopharyngeal swabs and 667 blood samples were gathered for SARS-CoV-2 testing.

The overall tested population consisted of 352 males (352/687, 51.24%) and 335 females (335/687, 48.76%). The most represented age groups were 40–49 years (126/687, 18.34%) and 50–59 years (121/687, 17.61%).

Out of 681 nasopharyngeal swabs collected, only one individual tested positive by qPCR (average CT 31.6). Seven people included in the survey had previously tested positive by qPCR at the beginning of March, but at the time of our study, they had already resulted negative to two consecutive nasopharyngeal swabs. As such, they had already been declared fully recovered from the disease before our study, and were no longer under health surveillance. All nasopharyngeal swabs taken form the seven previously confirmed cases tested negative. 

In contrast, out of the 667 sampled, 73 individuals, including the seven previously confirmed cases, tested positive for SARS-CoV-2 antibodies. The overall serological prevalence was 10.9% (95% CI, 8.8%–13.5%): 11.1% (95% CI, 8.2%–14.8%) for male and 10.8% (95% CI, 7.9%–14.7%) for female. No statistically significant difference was observed in the prevalence of infection between males and females (χ^2^ = 0.013 *p*-value = 0.90).

Concerning the eleven family groups in which one or more components tested positive for antibodies against SARS-CoV-2, the number of members was between two and six. In four out of the 11 groups, all family members tested positive for SARS-CoV-2 antibodies. In the remaining seven groups, only some individuals of the same household showed positive results. Statistical analysis indicated a statistical association between number of members of the household and positive result to the serological test (χ^2^ = 6.62 *p*-value = 0.037). 

Only one of the 19 tested children (from 0 to 9 years old) resulted positive for SARS-CoV-2 antibodies. The child lived together with parents, who tested negative. On the contrary, of the other negative 18, three were part of families that had at least one positive member. 

**Figure 1:**
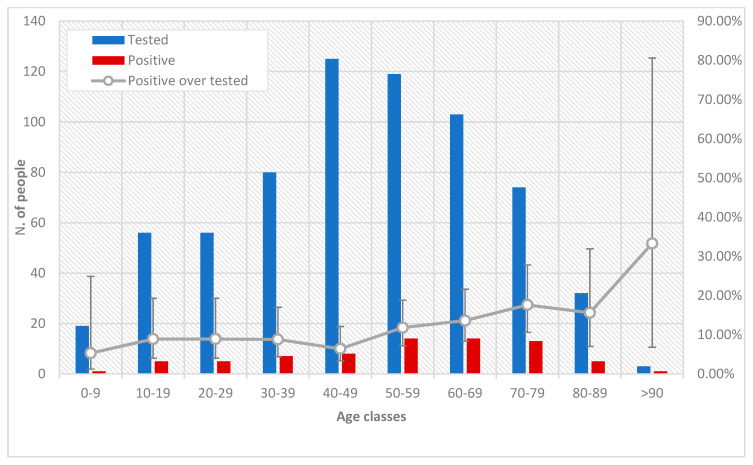
Individuals tested, and tested positive, for SARS-CoV-2 antibodies, stratified by age groups.

There were no significant differences among the serological prevalence in the different age groups (*p*-value = 0.148, Fisher’s exact test). Although individuals older than 50 years showed a twofold level of seroprevalence of infection than the lower age groups, these differences were not statistically significant (Figure 1). However, the median age of seropositive persons was 58.6 years (±28.5 years of IQR), significantly higher (*p*-value = 0.005, two tails Mann—Whitney Test) than that of negative individuals (median age: 47.3 years ± 28.1 years of IQR).

The frequency of clinical symptoms experienced by individuals during the two months prior to sampling was recorded. The 25.9% (95% CI, 16.4%–38.4%) of symptomatic individuals tested positive, versus the 9.5% (95% CI, 7.4%–12.1%) of the asymptomatic. The difference between the seroprevalence of infection in asymptomatic and symptomatic individuals was significant (χ^2^ = 14.50 *p*-value = 0.0001). 

Moreover, eight individuals declared to have been in close contact with a confirmed case, but only two of them resulted serologically positive.

Clear and unambiguous information on occupation was collected for 610 persons: 78 were classified as performing a job at risk for COVID-19, whereas the other 532 was considered not at risk. The “at risk” group included all the individuals with one or more of the following conditions: contact with the public (e.g., shop assistants, cashiers); physical proximity to other people (e.g., beauticians); and exposure to diseases and infections (e.g., health workers). All the other occupations (e.g., housewives and farmers) were considered to be “not at risk”. No difference, however, was observed between the serological prevalence in the two occupational categories (χ^2^ = 1.140 *p*-value = 0.286). 

Given the positive results to SARS-CoV-2 antibodies, 73 people were asked to submit additional information at a second epidemiological investigation round, which took place on 27 June. Sixty-five persons showed up for the survey. Of this second study group, 50 (50/65, 76.92%) reported clinical symptoms in the previous months. In 28 cases, the patient with symptoms consulted a doctor or went to the hospital seeking treatment. The most experienced symptoms were fatigue and/or muscle pain, fever (greater than 37.5 °C) and anosmia (Table 1).

When comparing the information collected in the two investigations regarding the presence of symptoms, 35 people provided inaccurate information in the first round, when they did not report to have experienced symptoms compatible with COVID-19.

**Figure 2:**
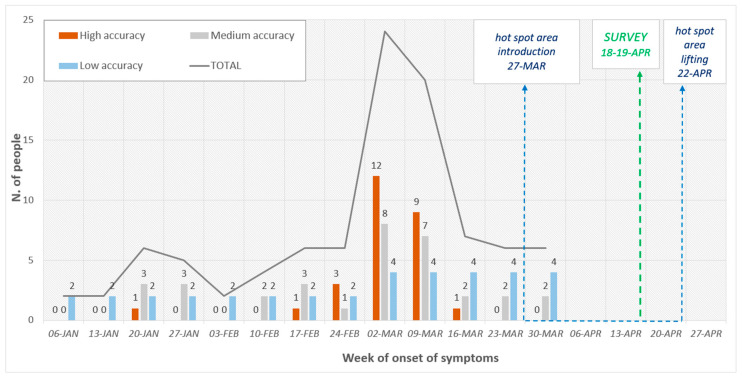
Week of onset of symptoms per accuracy of the information.

The onset date of symptoms was also investigated: 27 people provided this information with high accuracy (e.g., “01/03/2020”), 16 with medium accuracy (e.g., “at the beginning of March”) and seven with low accuracy (e.g., “March”). The given information was gathered per week (considering week starting from Monday): The highest (and most accurate) values of symptoms’ onsets were observed during the first two weeks of March 2020 (Figure 2). 

Out of the 65 people further investigated since testing positive to SARS-CoV-2 antibodies, 40 (40/65, 61.5%) had contact with a confirmed positive COVID-19 case, and 53 (53/65, 81.5%) stated that they used to regularly visit gathering/meeting areas (e.g., restaurants, places of worship, etc.) (Table 2), and the majority of the latter (37/53, 69.81%), more than once a week (Table 2). 

In addition to the familial clusters already identified during the first investigation, an additional cluster linked to a family-run bar and tobacco shop was recognized, in which 14 serologically positive persons (14/65, 21.54%) were involved: Five individuals were relatives of the owner (a confirmed case and the first COVID-19 victim in Abruzzo region), and nine persons were regular visitors of his business. 

## 4. Discussion

The survey conducted in Villa Caldari in April 2020 represented a great opportunity to study the transmission patterns of SARS-CoV-2 in a steady population, as the inhabitants of the village were confined at home for 22 days before the first survey (27 March–18 April). At the time of the sampling, 44 days after the first COVID-19 case in the village, twelve confirmed cases were known amid the population. This study was not meant to investigate the risk factors of SARS-CoV-2 infections, but rather to further explore the extent of the virus spread in the village and inform the Regional Authorities

In terms of RNA detection, only one case was identified through our study. This suggests that, at the time of the sampling, the virus was no longer actively circulating in the hotspot area. The patient, a woman who was approximately 80 years old, was placed under home-based health surveillance. Since she also tested positive to SARS-CoV-2 antibodies, she was included in the second survey population. On that occasion, she described having experienced some clinical symptoms, such as anosmia, dyspnoea, fever, dry cough, sore throat and fatigue. The new confirmed case, similarly to several other interviewed people, reported symptoms during the second investigation only, about two months after the first. This suggests some hesitancy to provide information on their health status and suggests to carefully interpret any conclusion based upon these data. 

The serological prevalence observed in Villa Caldari was 10.9% (95% CI, 8.8%–13.5%), which is four times greater than the value (2.5%) reported as preliminary result of a the nation-wide Italian survey on SARS-CoV-2 antibodies [17]. The prevalence observed in the present study was also higher than the one estimated for the overall Lombardy region, the most heavily affected region by the pandemic in Italy, which stood at 7.5% [17]. At the same time, it was considerably lower than the serological prevalence observed in two municipalities severely affected by the pandemic, Bergamo (24%) and Cremona (19%) [17]. A more similar prevalence was observed in Central Spain and in the area of Madrid (>10%) [18], although transmission dynamics in a small village like Villa Caldari could be very different from what expected in metropolitan areas, where public transportations and other places with a high concentration of people can play a major role in the spread of the infection. In a small village, other aggregative sites, such as bars, pubs, shops, etc., together with the intense social relationships among relatives, may place a crucial role in the transmission of the infection, as demonstrated by the numerous familial clusters identified by the present study, as well as the cluster linked to a bar and tobacco shop. Villa Caldari is a small town, and close contacts between families could be assumed. The transmission of the infection within households was highlighted in this report, where contact occurs over a prolonged period and in close proximity: in four out of the 11 investigated households, all the family members tested positive for SARS-CoV-2 antibodies. Familial clusters in which the viral shedding of the virus occurred at the early stages of infection and in the absence of symptoms have already been observed [19,20]. Moreover, a very high odd ratio to become infected was observed when living together with a confirmed case [19]. Moreover, one more cluster was linked to typical social behaviors: at least 14 people were connected to a family-run bar and tobacco shop, as regular customers or relatives of the owner. This is not a new situation, as COVID-19 proved to be commonly transmitted by the close contact of people, and clusters due to gatherings were reported [20]. However, it is beyond the scope of our study to investigate where the contagion started and define the transmission chain. 

At present, very limited studies conducted in small hotspot areas are available. The sole published study regarding Italy is the one by Lavezzo et al. [19]. In this study, the authors performed a serological survey in a small town (Vò Euganeo) of about 3200 inhabitants, located in Veneto region (Northeastern Italy), observing a prevalence of infection of 2.6% (95% CI, 2.1%–3.3%), which is largely lower than that observed in Villa Caldari. This difference can be explained by the different time in the epidemic on which the two serological surveys were performed. In the case of Vò Euganeo, the survey was conducted around the time the town lockdown started, quite early in the emergence of the infection, whereas we carried out our study 44 days after the first case in the village and 22 days after the lockdown was put in place. 

Nevertheless, the serological prevalence observed in Villa Caldari confirms that, even in areas with intense virus circulation, most of the population has remained unexposed to SARS-CoV-2 [21,22]. In light of this, the achievement of herd immunity without accepting the burden of collateral damage, as the overload of health systems and fatalities in the susceptible population, would be difficult to reach, even in small populations [18]. 

In line with the preliminary results of the Italian nation-wide study [17], no significant difference of serological prevalence between genders was observed in Villa Caldari. Only one out of 19 children tested positive to the antibodies, and a lower prevalence of infection in children has been reported elsewhere [18,21]. In our study, 15% of children testing negative to the antibodies belonged to families of which some members also tested positive to SARS-CoV-2 antibodies. This finding is in contrast with the general observation of the importance of familiar clusters in Villa Caldari, and it could suggest a lower susceptibility of children to infection, although a controversial debate is ongoing in the scientific community on the epidemiological role of youngsters and children in the transmission of the virus. More simply, what observed could be the effect of an increased awareness in the people about the risks of SARS-CoV-2 transmission during the lockdown phase, with a strict respect of quarantine measures in the households.

Despite the pre-existing evidence that some jobs are at a greater risk for SARS-CoV-2 infection (e.g., healthcare workers), people involved in this study who have an occupation considered at risk did not show any significant difference in terms of positivity to SARS-CoV-2 antibodies. 

Similar to the results of the Italian-based study [17], almost one-fourth of people who tested positive for SARS-CoV-2 antibodies did not report any clinical symptom. Approximately one-third of seropositive participants were asymptomatic in the Spanish-based study [18]. The high proportion of asymptomatic infections has important public health implications, given the potential key role in the transmission of the virus by asymptomatic individuals [18,19].

Amongst the symptomatic infections, the most frequent symptoms are consistent with COVID-19 [23,24]. In our population, dyspnoea was poorly reported, possibly because outpatients not presenting severe respiratory distress composed it [25]. Anosmia (the loss of the ability to detect one or more smells) should be recognized as an early symptom of COVID-19 [26], and therefore specifically addressed in surveillance activities. Except for the patients that consulted a doctor or went to the hospital seeking treatment, we could assume that the rest experienced mild-to-severe symptoms. Serological testing was also able to detect people with mild or asymptomatic disease who would have recovered by the time of sampling, allowing for a more accurate estimate of infected cases in the population. 

Most of the population of the second survey reported the onset of symptoms within the first two weeks of March, with different levels of accuracy. The hotspot measures were implemented on 27 March, when the largest part of people already had or was experiencing symptoms. This finding provides further confirmation that SARS-CoV-2 may have been circulating since long before the Italian lockdown, as shown by its presence in Italian wastewaters since December 2019 [27]. 

Considering only a few people reported an onset of symptoms later during the month, it may be assumed that the lockdown measures were effective in breaking the chain of transmission and prevented new cases. A similar situation was described by Lavezzo and colleagues [19]: the early identification and isolation of cases, in association with community lockdown, was effective in the suppression of viral transmission in Vo’ Euganeo, one of the first COVID-19 hotspots in Italy. Therefore, it can be concluded that the prompt application of social restriction measures, coupled with the identification of infectious individuals through the extensive testing of (large part of) the population, represent the pillars for hampering the transmission of the virus, reducing the impact of the disease and, eventually, allowing an earlier withdrawal of the lockdown measures and a quickest recovery from the social and economic impacts of its application.

## 5. Conclusions

The detected serological prevalence highlighted how, in a small village with many interactions, SARS-CoV-2 circulation was high. Nevertheless, the interventions implemented in Villa Caldari showed a substantial reduction of SARS-CoV-2 circulation in the village and demonstrated that COVID-19 suppression can be rapidly achieved after applying severe control measures. Thoroughly testing the population, wherever feasible, permits us to identify residual sources of infection and effectively stop the contagion. 

With the present study, it was possible to identify only one individual who tested positive for SARS-CoV-2 RNA. Serological testing was able to detect a prevalence of 10.9% (95% CI, 8.8–13.5%): No significant difference of serological prevalence between genders and in different age groups was observed, while the difference between the seroprevalence of infection in asymptomatic and symptomatic individuals was significant. The serology survey also made it possible to detect people with mild or asymptomatic disease, allowing for a more accurate estimate of infected cases in the population.

Overall, the epidemiological situation was therefore favorable for the lifting of the hotspot emergency measures, which were suspended with effect from 22 April [28]. 

## Figures and Tables

**Table 1:** Reported symptoms by the second study group (number of people showed up for the second survey: 65).

Symptom	N. of People	Percentage in the Second Study Group
Fatigue/muscle pain	32	49.23%
Fever (>37.5 °C)	30	46.15%
Anosmia	21	32.31%
Dry cough	17	26.15%
Diarrhea/vomit	14	21.54%
Sore throat	12	18.46%
Headache	12	18.46%
Dyspnoea	5	7.69%
Other symptoms*	4	6.15%
Conjunctivitis	1	1.54%

* Additional symptoms were reported beyond those specifically investigated: discomfort (n. 1), cold (n. 1) and intercostal pain (n. 2).

**Table 2:** Information collected during the surveys from people tested positive for SARS-CoV-2 antibodies (number of people showed up for the second survey: 65).

Data from the Questionnaires	Possible Answers	Household Size (*n*. Components)						Total
		*1*	*2*	*3*	*4*	*5*	*8*	
Contact with a confirmed COVID-19 case	Yes	11	11	12	2	4		40
	No	5	1	5	1	1	6	19
	I do not know/no answer	4		1	1			6
Health professional	Yes	1	1					2
	No	19	11	18	4	5	6	63
Occupation permitted during the Italian lockdown	Yes	2	3				1	6
	No	18	9	18	4	5	5	59
Attendance of gathering places (restaurants, bars, places of worship, etc.)	Yes (no specification)	1						1
	Yes, once a week	1	1	3	1	3	1	10
	Yes, more than once a week	10	8	13	3	2	1	37
	Yes, a couple a month	1	1				1	3
	Yes, once every one or two months	1		1				2
	No	6	2	1			3	12
Positive to previous RT-PCR for SARS-CoV-2	Yes	6		1				7
	No	14	12	17	4	5	6	58

## Supplementary Materials

The following are available online at https://www.mdpi.com/2076-2607/8/11/1711/s1.
