# Supplementary material for: A COVID-19 Hotspot Area: Activities and Epidemiological Findings
Source: Microorganisms. 2020 Oct 31;8(11):1711. doi: 10.3390/microorganisms8111711 (PMC7692759; doi:10.3390/microorganisms8111711)
Supplement: Supplementary file 1 [file microorganisms-08-01711-s001.zip › Supplementary files/Questionnaire_survey_1.pdf]

# EPIDEMIOLOGICAL STUDY ON THE SPREAD OF SARS-COV-2

N. of registry

| **APPLICANT:**<br>EMAIL<br>PHONE | DATE | TIME |
|---|---|---|

**TYPE OF SAMPLE:** ☐ SWAB ☐ SERUM

**PATIENT*:** ☐ COVID-19 SUSPECTED ☐ COVID-19 CONFIRMED

☐ CONTACT WITH CONFIRMED/SUSPECT COVID-19 CASE ☐ HOSPITALIZED PATIENT

X UNDER HEALTH SURVEILLANCE ☐ NONE OF THE ABOVE

***It is possible to select more than one option**

LAST NAME: ……………………………………… FIRST NAME: …………………………………………………

SOCIAL SECURITY NUMBER:………………………………………………………………………………………………

SEX: ☐ M ☐ F DATE OF BIRTH: ………………………………………………

PHONE/MOBILE NUMBER: ………………………………………………………………………………………………

ADDRESS:…………………………………………………………………

MUNICIPALITY:………ORTONA (CH)……………………………………………………………… ……………

OCCUPATION: …………………………………………………………………………………………………………

FAMILY NUMBER (FIRST NAME, LAST NAME, DATE OF BIRTH):

1) ……………………………………………………………………………… _ _ / _ _/ _ _ _ _

2) ……………………………………………………………………………… _ _ / _ _/ _ _ _ _

3) ……………………………………………………………………………… _ _ / _ _/ _ _ _ _

4) ……………………………………………………………………………… _ _ / _ _/ _ _ _ _

5) ……………………………………………………………………………… _ _ / _ _/ _ _ _ _

6) ……………………………………………………………………………… _ _ / _ _/ _ _ _ _

## CLINICAL EVALUATION

IN THE LAST SEVEN DAYS, DID YOU EXPERIENCE ONE OR MORE OF THE ABOVE SYMPTOMS:

| | | | |
|---|---|---|---|
| FEVER | ☐ | SORE THROAT | ☐ |
| DISCOMFORT/FATIGUE | ☐ | DYSPNOEA | ☐ |
| HEADACHE | ☐ | VOMITING/DIARRHEA | ☐ |
| ARTHRALGIA | ☐ | COUGHING | ☐ |

DATE OF ONSET OF SYMPTOMS: _ _ / _ _/ _ _ _ _

## EPIDEMIOLOGICAL INVESTIGATION

ARE YOU A HEALTHCARE PROFESSIONAL AND/OR DID YOU WORK OR GO TO A MEDICAL FACILITY WHERE COVID-19 PATIENTS ARE HOSPITALIZED?

☐ YES ☐ NO

IF YES, WHERE? ……………………………………………………………………………………………………………

IS YOUR OCCUPATION CLASSIFIED AS “ESSENTIAL”, AND THEREFORE PERMITTED DURING THE ITALIAN LOCKDOWN (DPCM 11/03/2020)?

☐ YES ☐ NO

IF YES, COULD YOU PLEASE INDICATE YOUR PLACE OF WORK?
…………………………………………………………………………………..

ARE YOU A PET OWNER? ☐ YES ☐ NO

IF YES, WHICH ONE/ONES? ☐ DOGS (N __ ) ☐ CATS (N __ )

IS/ARE YOUR PET/PETS LIVING

DOG : ☐ ALWAYS INSIDE AT HOME ☐ ALWAYS OUTDOOR ☐ AT HOME AND OUTDOOR

CAT : ☐ ALWAYS INSIDE AT HOME ☐ ALWAYS OUTDOOR ☐ AT HOME AND OUTDOOR

**PRIORITY LEVEL** : ☐ RED ☐ YELLOW ☐ GREEN

FIRST NAME AND LAST NAME OF THE REQUESTING DOCTOR: ..............................................................................................

SIGNATURE: ___________________________________________

FIRST NAME AND LAST NAME OF THE NURSE THAT DID THE SAMPLING: ……...……………………………………

SIGNATURE: ___________________________________________

---

*I hereby authorize the processing of my personal data in accordance with Reg EU/2016/679, art. 13 and 14.*

*☐ YES ☐ NO*

*Signature* ______________________
