# Supplementary material for: A COVID-19 Hotspot Area: Activities and Epidemiological Findings
Source: Microorganisms. 2020 Oct 31;8(11):1711. doi: 10.3390/microorganisms8111711 (PMC7692759; doi:10.3390/microorganisms8111711)
Supplement: Supplementary file 1 [file microorganisms-08-01711-s001.zip › Supplementary files/Questionnaire_survey_2.pdf]

SEROLOGICAL STUDY ON SARS-COV-2 SPREAD IN VILLA CALDARI, ORTONA (CH)

EPIDEMIOLOGICAL INVESTIGATION

ID. N.:
____

PERSONAL DATA
LAST NAME: ………………………………………. FIRST NAME……………………………………………
SOCIAL SECURITY NUMBER …………………………………………………………………
SEX: ☐ M ☐ F DATE OF BIRTH………………………………………………
PHONE/MOBILE NUMBER: ………………………………………………………………………………………
ADDRESS: ………………………………...
MUNICIPALITY………ORTONA (CH)…………..…………………………………………………………………
OCCUPATION: ……………………………………………………………………………………………......

I hereby authorize the processing of my personal data in accordance with Reg EU/2016/679, art. 13 and 14.

☐ YES ☐ NO

Signature _______________________

CLINICAL EVALUATION

SINCE THE 1ST OF FEBRUARY, HAVE YOU EXPERIENCED ANY OF THE FOLLOWING SYMPTOMS?

SYMPTOM	NO	YES	IF YES, PLEASE SPECIFY DATE OF ONSET	IF YES, FOR HOW MANY DAYS?	IS IT STILL PRESENT? (YES/NO)	I DO NOT REMEMBER/KNOW
ANOSMIA						
CONJUNCTIVITIS						
VOMITING/DIARRHEA						
DYSPNOEA						
FEVER (>37.5^{O}C)						
SORE THROAT						
HEADACHE						
FATIGUE / MUSCLE PAIN						
DRY COUGH						

DURING THE ILLNESS:

Did you consult a doctor?	☐ YES ☐ NO ☐ I DON'T KNOW
Did you go to the Emergency Room?<br><br>If yes, to which hospital?	☐ YES ☐ NO ☐ I DON'T KNOW<br><br>……………………………………………………………
Have you been hospitalized because of the illness?<br><br>If yes, in which hospital?	☐ YES ☐ NO ☐ I DON'T KNOW<br><br>………………………………………………………………
Have you been under health surveillance?<br><br>If yes, from when to when?<br><br>And why?	☐ YES ☐ NO ☐ I DON'T KNOW<br><br>……………………………- ………………………………<br><br>o Positive swab<br>o Contact of a confirmed case<br>o Other reasons…………………………………
Did you undergo to one or more swab for SARS-CoV-2 detection?	☐ YES ☐ NO ☐ I DON'T KNOW

**Date** (sampling)	**Place** (hospital, health surveillance, workplace, etc.)	**Result**

ANY OF YOUR ACQUAINTANCES OR COLLEAGUE, EXPERIENCES SIMILAR SYMPTOMS IN THE REFERRED PERIOD?

☐ YES ☐ NO ☐ I DON'T KNOW

STARTING FROM THE 1ST OF FEBRUARY, DID YOU HAVE ANY CONTACT WITH CONFIRMED COVID-19 CASES?

☐ YES ☐ NO ☐ I DON'T KNOW

- IF YES, PLEASE SPECIFY:

☐ RELATIVES – SAME HOUSEHOLD

☐ RELATIVES – DIFFERENT HOUSEHOLD

☐ WORK COLLEAGUES / CLASSMATES

☐ FRIENDS

☐ OTHER :………………………………………………………………………………………………………

EPIDEMIOLOGICAL INVESTIGATION

ARE YOU A HEALTH PROFESSIONAL? ☐ YES ☐ NO

- IF YES, WHAT IS YOUR ROLE? ……………………………………………………………………………………......
- IF YES, DID YOU WORK OR GO TO MEDICAL FACILITIES WHERE COVID-19 PATIENTS ARE HOSPITALIZED?

☐ YES ☐ NO ☐ I DON'T KNOW

- IF YES, WHERE? ………

IS YOUR OCCUPATION CLASSIFIED AS "ESSENTIAL", AND THEREFORE PERMITTED DURING THE ITALIAN LOCKDOWN (DPCM 11/03/2020)?

☐ YES ☐ NO ☐ I DON'T KNOW

- IF YES, COULD YOU SPECIFY YOUR PLACE OF WORK ………………………………………………………..
- IF YES, COULD YOU SPECIFY YOUR ROLE……………………………………………………………………..

DURING YOUR WORK, ARE YOU IN CONTACT WITH THE PUBLIC?

☐ YES ☐ NO ☐ I DON'T KNOW

SINCE THE 1ST OF JANUARY, HAVE YOU BEEN TO GATHERING PLACES (RESTAURANTS, BARS, PLACES OF WORSHIP, etc.)

☐ YES ☐ NO ☐ I DON'T KNOW

IF YES, HOW OFTEN?

- MORE THAN ONCE A WEEK
- ONCE A WEEK
- TWICE A MONTH
- ONCE EVERY MONTH OR TWO

SINCE THE 1ST OF JANUARY, DID YOU DO TRAVEL OUTSIDE YOUR REGION?

- IF YES, FROM WHERE AND TO WHERE? …………………………………………………………
- IF YES, FOR WHICH REASON? (i.e. work, tourism, familiar issues, study, etc.)

………..

NOTES AND OBSERVATIONS

____

____

____

____

____

____

____

____

____

____

____
