# Supplementary figures and images for: A COVID-19 Hotspot Area: Activities and Epidemiological Findings
Source: Microorganisms. 2020 Oct 31;8(11):1711. doi: 10.3390/microorganisms8111711 (PMC7692759; doi:10.3390/microorganisms8111711)

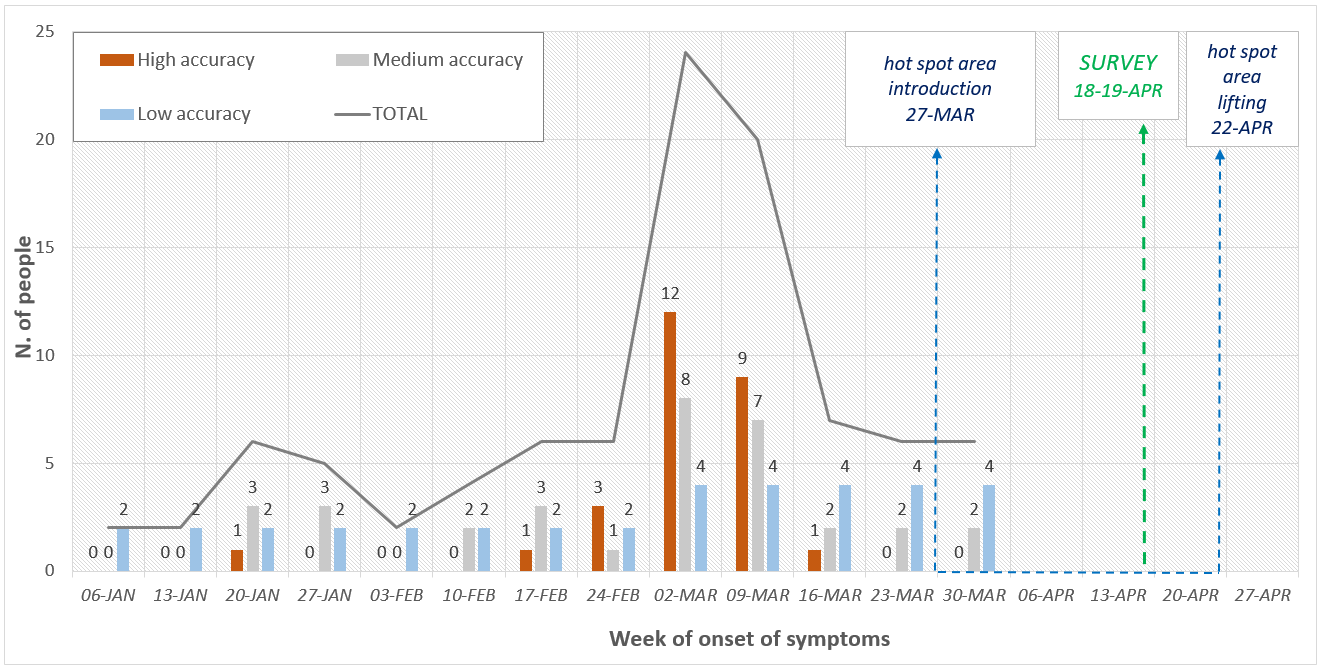

Supplement: Supplementary file 1 [file microorganisms-08-01711-s001.zip › Supplementary files/Figure2.PNG]
